# A genome-wide association study of deafness in three canine breeds

**DOI:** 10.1371/journal.pone.0232900

**Published:** 2020-05-15

**Authors:** Jessica J. Hayward, Maria Kelly-Smith, Adam R. Boyko, Louise Burmeister, Luisa De Risio, Cathryn Mellersh, Julia Freeman, George M. Strain

**Affiliations:** 1 Department of Biomedical Sciences, College of Veterinary Medicine, Cornell University, Ithaca, New York, United States of America; 2 Department of Comparative Biomedical Sciences, School of Veterinary Medicine, Louisiana State University, Baton Rouge, Louisiana, United States of America; 3 Animal Health Trust, Newmarket, Suffolk, England, United Kingdom; Faculty of Animal Sciences and Food Engineering, University of São Paulo, BRAZIL

## Abstract

Congenital deafness in the domestic dog is usually related to the presence of white pigmentation, which is controlled primarily by the *piebald* locus on chromosome 20 and also by *merle* on chromosome 10. Pigment-associated deafness is also seen in other species, including cats, mice, sheep, alpacas, horses, cows, pigs, and humans, but the genetic factors determining why some piebald or merle dogs develop deafness while others do not have yet to be determined. Here we perform a genome-wide association study (GWAS) to identify regions of the canine genome significantly associated with deafness in three dog breeds carrying *piebald*: Dalmatian, Australian cattle dog, and English setter. We include bilaterally deaf, unilaterally deaf, and matched control dogs from the same litter, phenotyped using the brainstem auditory evoked response (BAER) hearing test. Principal component analysis showed that we have different distributions of cases and controls in genetically distinct Dalmatian populations, therefore GWAS was performed separately for North American and UK samples. We identified one genome-wide significant association and 14 suggestive (chromosome-wide) associations using the GWAS design of bilaterally deaf vs. control Australian cattle dogs. However, these associations were not located on the same chromosome as the *piebald* locus, indicating the complexity of the genetics underlying this disease in the domestic dog. Because of this apparent complex genetic architecture, larger sample sizes may be needed to detect the genetic loci modulating risk in piebald dogs.

## Introduction

The most common form of deafness in dogs is hereditary, congenital, and associated with white pigmentation [1–3]. The most associated genetic locus in dogs is *piebald* [4]. Several studies have shown that a strong melanocyte suppression by recessive alleles, evidenced by blue irises and absence of pigmentation in the tapetum lucidum, is positively associated with early postnatal deafness, while weak melanocyte suppression, as evidenced by patches (solid colored areas larger than spots, located anywhere on the body, colored black or liver) in Dalmatians and facial masks and body marks in Australian cattle dogs, is negatively associated with deafness [5–7]. However, the inheritance of this deafness does not follow simple Mendelian inheritance patterns (reviewed by [1]). Pigment-associated hereditary deafness is observed in numerous other species, most frequently recognized in blue-eyed white cats [1,3,8,9].

The genetic basis of the *piebald* locus has been shown to be a SINE insertion in a 3.5-kb region upstream of the *M* promoter region of the gene *MITF* (*melanogenesis associated transcription factor*, formerly *microphthalmia-associated transcription factor*), located on canine chromosome CFA20 [10,11,12]. The protein encoded by the *MITF* gene is a transcription factor that contains both basic helix-loop-helix and leucine zipper structural features, and regulates melanocyte development and is responsible for pigment cell-specific transcription of the melanogenesis enzyme genes. Mutations are known to cause the human pigmentary deafness syndromes Waardenburg Syndrome type IIa and Tietz Syndrome [12], and mutations of the gene have been shown to cause deafness in several species: horse [13], cow [14], pig [15], mink [16], and mouse [17].

*MITF* is described as the master transcriptional regulator of pigmentation, and more than 125 distinct genes are known to directly or indirectly regulate pigmentation [18,19]. *MITF* is involved in melanocyte differentiation, melanocyte cell-cycle progression and survival, and in the induction of melanomas. These actions are mediated through (1) transcription factors such as PAX3, SOX10, TCF, LEF-1, and CREB; (2) upstream signaling factors such as WNT acting on FZD and then β catenin, αMSH acting on MC1R and then cAMP, and EDN3 acting on EDNRB and KITLG acting on KIT, both then acting through (3) the posttranslational signaling pathway factor MAPK [3,19]. MITF exists as at least six different isoforms in dogs (A, D, E, H, J, and M), but only the M isoform is present in melanocytes [20]. There is incredible complexity in the numerous gene factors acting on MITF and the gene products that MITF acts upon. Recent proteomic studies of the cochlea [21,22] provide a catalog of genes expressed in these tissues to guide molecular genetic studies.

Histologic studies of cochlear structures have shown that pigment-associated deafness is the result of the primary degeneration of the stria vascularis, a structure on the outer cochlear duct. Cochlear hair cells die subsequent to the strial degeneration. The stria is a three-cell thick structure that functions in a similar manner to the blood-brain barrier [23], preventing passage of all but small lipophilic molecules into the cochlear duct endolymph. The stria also secretes potassium ions, necessary for hair cell depolarization, into the endolymph that surrounds hair cell stereocilia [24]. The strial layers consist of endolymph-facing marginal cells, intermediate cells (melanocytes), and basal cells [25]. The intermediate cells–melanocytes–function not to produce melanin but to participate in the potassium cycle.

It is known from canine histologic studies that deaf cochleae have a degenerated stria, but the cause and mechanism of degeneration are not known. Melanocyte precursors migrate in the embryo from the neural crest to the cochlea where they develop from melanoblasts into functional melanocytes, a process involving multiple transcription factors and other signaling molecules [26,27,28]. Recent studies of a naturally occurring pigment-associated deafness disorder in Rongchang pigs caused by a *de novo* silencer mutation in *MITF-M* expression [15] documented the embryonic and postnatal development of the stria [28]. They showed that a normal stria vascularis did not develop in *MITF*^–/–^pigs, normally present in embryonic day 85 (E85) pigs, but instead consisted of a thin, loose collection of cells that included intermediate cells in affected pigs, followed by collapse of the cochlear duct by E100, followed by degeneration of spiral ganglion neurons by postnatal day 30. Intermediate cells (melanocytes) were present at E85 but the stria never developed its mature histologic pattern.

Canine deafness studies have not identified genes in proximity to significant GWAS SNPs or linkage markers that had previously been identified as causative in human or mouse deafness disorders. Here, we undertook an association study using SNP data from across the entire canine genome to investigate the genetic cause of pigment-associated deafness in three piebald dog breeds: the Dalmatian, Australian cattle dog, and English setter.

## Materials and methods

### Subjects

North American samples were collected with informed consent under approved LSU Institutional Animal Care and Use Committee protocol 15–104. AHT samples were collected with informed consent by buccal swabbing, a non-invasive technique that does not require a UK Home Office license. Samples were sourced Nov 2014 to Feb 2018 from North America through US breed clubs and clients seen at Louisiana State University School of Veterinary Medicine, and from the UK through the Animal Health Trust (AHT). From North America, we collected 106 Australian cattle dog, 123 Dalmatian, and 65 English setter saliva samples. In addition, 61 DNA samples from 22 Dalmatian litters were received from the Orthopedic Foundation for Animals (OFA; www.offa.org), to give a total of 184 North American Dalmatian samples. For UK (AHT) samples, 14 Australian cattle dog, 120 Dalmatian, and 14 English setter saliva or DNA samples were included.

### DNA collection and isolation

DNA samples from hearing and affected dogs were collected in accordance with Louisiana State University animal care and use guidelines (LSU IACUC 15–104). North American saliva samples were collected with informed consent into Performagene PG-100 sample vials (DNA Genotek) by the investigators at the time of hearing testing, or by dog owners, using a process that does not produce stress or distress. AHT samples were collected with informed consent using buccal swabs. DNA was isolated from the saliva using the prepIT-L2P kit (DNA Genotek) and frozen at -20°C for later analysis.

### Hearing assessment

Hearing status was confirmed by means of the brainstem auditory evoked response (BAER) hearing test [29]. Tests were performed by one North American investigator (GMS), by AHT investigators (JF, LDR), or for the samples provided by owners, a copy of a verified BAER test result was required to confirm hearing status. From the BAER test, subjects were classified as bilaterally hearing, unilaterally deaf, or bilaterally deaf. Phenotypic data on eye color or presence of patches was not available for most subjects, and so was not considered.

### Genotyping and population structure

DNA samples were genotyped on the semi-custom 220k CanineHD array (Illumina). Quality control was performed in PLINK v 1.90 [www.cog-genomics.org/plink/1.9/, 30]. Samples with >3% missingness were removed from analysis, SNPs with >95% missingness were removed, and SNPs that deviated from Hardy-Weinberg equilibrium (observed:expected heterozygosity ratio of <0.25 or >1.0) were also removed. In total, we included 304 Dalmatians, 120 Australian cattle dogs, and 79 English setters genotyped at 201,020 SNPs. To look for population structure, a principal component analysis (PCA) was performed for each breed using the program EIGENSTRAT (EIGENSOFT v5.0.1 package) [31]. SNPs present at <5% minor allele frequency in each cohort were removed from that cohort’s analysis.

### Genome-wide association study

For each dog breed (Dalmatian, Australian cattle dog, English setter), we implemented a three-part GWAS design. First, we used a sibling-pair matched case/control design, in which we included a control (bilaterally hearing) for every case (either bilaterally or unilaterally deaf) from the same litter. We included 33 cases and 33 controls (from 33 litters) for the English setters, 52 cases and 52 controls (from 46 litters) for the Australian cattle dogs, 43 cases and 43 controls (from 38 litters) for the UK Dalmatians, and 82 cases and 82 controls (from 72 litters) for the North American Dalmatians. Due to the sampling design, we ran the sibling-pair analysis in PLINK without including a genetic relatedness matrix. By including a matched case for every control from each litter, we remove the need to control for underlying population structure because, on average, the only differences in common among these cases (compared to their sibling controls) should be SNPs that are involved in causing deafness.

Second, we used a quantitative GWAS design, where bilaterally deaf dogs were assigned a 1, unilaterally deaf dogs were assigned a 2, and bilaterally hearing dogs were assigned a 3, and there was no regard for relatedness. We included 11 deaf, 29 unilaterally deaf, and 39 control English setter dogs, 16 deaf, 42 unilaterally deaf, and 61 control Australian cattle dogs, 72 deaf, 5 unilaterally deaf, and 43 control UK Dalmatian dogs, and 20 deaf, 73 unilaterally deaf, and 91 control North American Dalmatian dogs. The quantitative design was run in the program GEMMA [32] using a relatedness matrix as a random effect in the linear mixed model and the Wald Test to calculate *P*-values. Third, in addition to this quantitative GWAS design, we also ran a case/control analysis in GEMMA using only bilaterally deaf and control dogs (that is, excluding the unilaterally deaf dogs) for each population. This analysis was performed to see if we could increase power by removing dogs with an intermediate phenotype.

Significance thresholds were set using the Bonferroni correction (alpha = 0.05) on unlinked SNPs (determined using—indep 100 10 10 in PLINK) for each analysis. Suggestive thresholds were set using the Bonferroni correction (alpha = 0.05) on unlinked SNPs at the chromosome level. For each analysis, we also randomly permuted the phenotypes (using—make-perm-pheno 10000 in PLINK) and reran the GWAS 10,000 times to determine the 5% *P*-value threshold. As these permutation thresholds were all smaller (more stringent) than the thresholds calculated on unlinked SNPs, we used the latter to determine genome-wide significance. Manhattan and QQ plots were generated in R [33] using qqman, LD plots were generated in Jupyter notebook using Matplotlib library [34,35].

## Results

### Population structure

**Fig 1:**
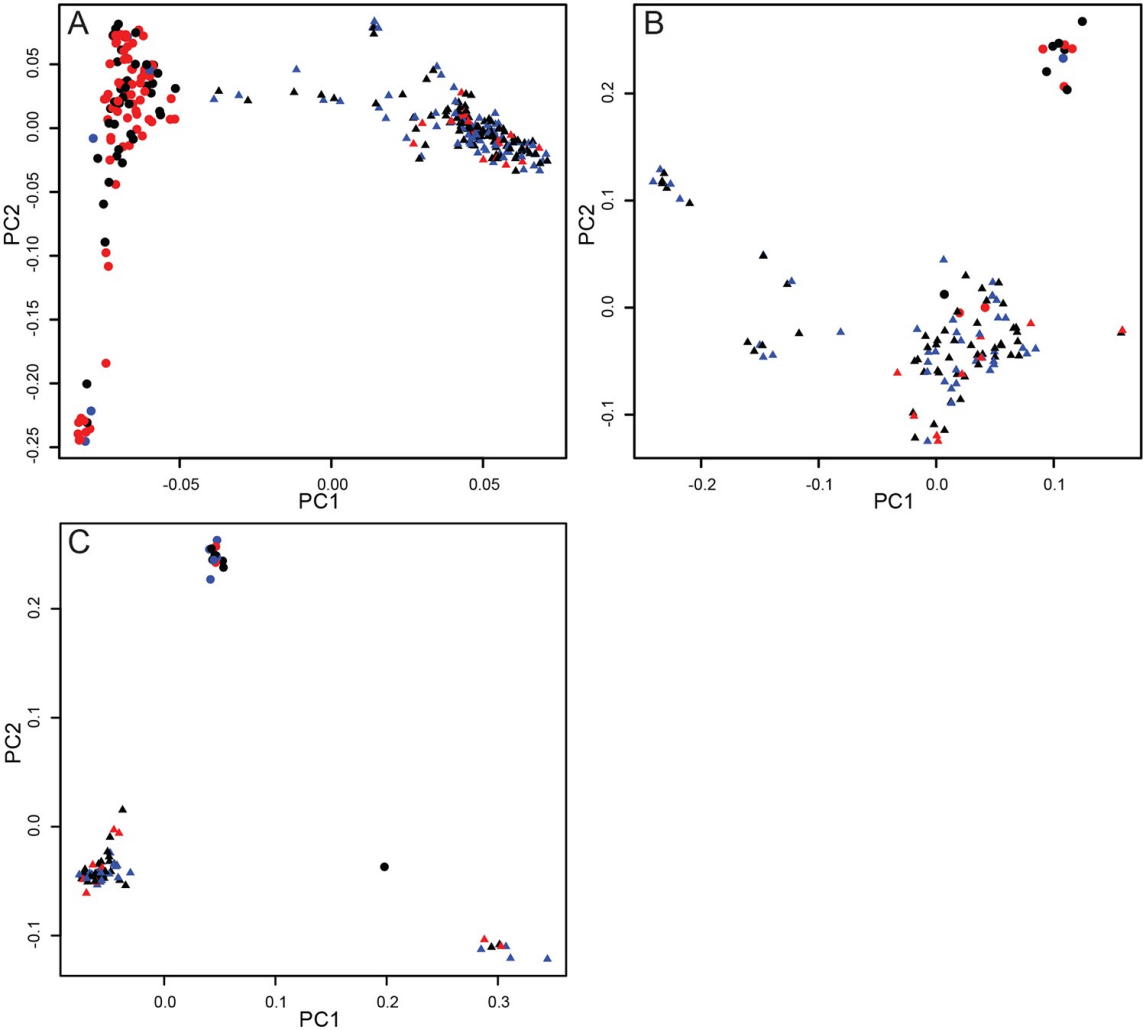
PCA of dog samples. (A) Dalmatians, (B) Australian cattle dogs, and (C) English setters from North America (triangles) and UK (circles). Bilaterally deaf dog samples are shown in red, unilaterally deaf dogs in blue, and control dogs in black.

PCA showed a genetic difference between the North American and UK populations of Dalmatians and Australian cattle dogs (Fig 1). For the Dalmatians, sample geographic origin separates on PC1 with two distinct clusters (Fig 1A). There are different phenotype patterns for the two geographic regions–North American samples are predominantly controls (50% of samples) and unilaterally deaf (40% of samples), while the UK samples are predominantly bilaterally deaf (60% of samples) and controls (36% of samples) (Fig 1A). Due to this different distribution of cases and controls in genetically distinct populations, Dalmatian dogs were separated into North American and UK samples for analyses.

In the Australian cattle dog (ACD) PCA, sample geographic origin separates on PC2, with the exception of three UK samples (26427, 17551, 26425) that grouped with the North American samples (Fig 1B). Given that we had so few ACDs from the UK (n = 14, 12% of all ACD samples) and there was no phenotype separation as seen in the Dalmatians, ACD GWAS was performed without excluding any samples.

We saw some geographic structure in the English setter PCA (Fig 1C) but, like the ACDs, we did not have a biased phenotypic distribution from the different locations, so all dogs were included in the GWAS.

### Genome-wide association study

**Fig 2:**
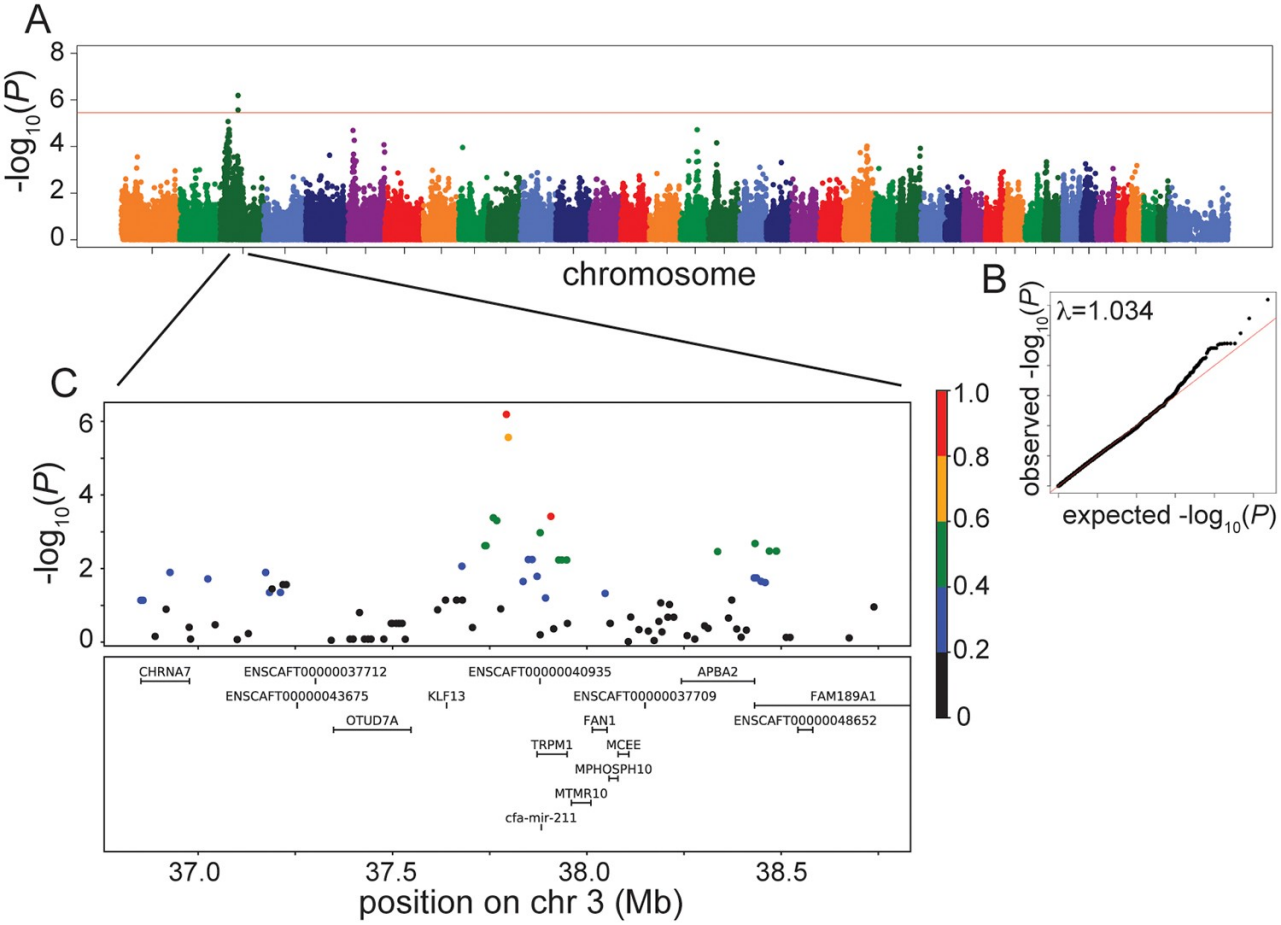
Manhattan, QQ and LD plots of the significant association using bilaterally deaf vs. control Australian cattle dogs. (A) Manhattan plot. Red line shows the Bonferroni correction *P*-value threshold calculated on unlinked SNPs. The permutation threshold is beyond the y axis, at 1.5x10^-9^. (B) QQ plot with inflation factor (λ) shown. (C) LD plot zoomed in on the region surrounding the significant association. Colors indicate the amount of LD with the most associated SNP, ranging from red (r^2^>0.8) to black (r^2^<0.2).

From all our different analyses using a sibling-pair GWAS design, a quantitative GWAS design, and a case-control (bilaterally deaf vs. controls) GWAS design, we identified only one significant association at the genome-wide level–that of bilaterally deaf vs control Australian cattle dogs (Table 1, S1 Table). This significant association consisted of two SNPs on CFA3 (3:37,793,043, *P* = 6.5×10^−7^ and 3:37,797,912, *P* = 2.7×10^−6^) that passed the Bonferroni threshold, calculated on unlinked SNPs, of 3.6×10^−6^ (Fig 2, S2 Table).

**Table 1:** Most significant SNPs identified through case-control (bilaterally deaf vs. controls) genome-wide association studies in Dalmatians, Australian cattle dogs, and English setters.

Breed	N	CFA	bp	SNP ID	af all (deaf/controls)	pve^a^	beta	*P*-value
Dalmatians North America	20 deaf, 91 controls	30	37,235,914	BICF2P1106247	0.104 (0.300/0.060)	0.167	-0.353	7.25×10^−6^*
		30	33,816,254	BICF2P113616	0.333 (0.625/0.269)	0.155	-0.222	1.60×10^−5^*
		23	48,506,877	BICF2G630365393	0.441 (0.725/0.379)	0.150	-0.220	2.28×10^−5^*
		30	22,647,163	BICF2G630405064	0.068 (0.200/0.038)	0.130	-0.408	8.93×10^−5^*
		37	27,255,309	BICF2G630132623	0.243 (0.450/0.198)	0.122	-0.245	1.54×10^−4^*
Dalmatians UK	72 deaf, 43 controls	38	21,626,523	BICF2G63068103	0.152 (0.083/0.267)	0.127	0.350	8.22×10^−5^*
Australian cattle dogs	16 deaf, 61 controls	3	37,793,043	BICF2G630338450	0.299 (0.656/0.205)	0.277	-0.313	6.46×10^−7^**
		3	17,067,881	BICF2G630703558	0.117 (0.344/0.057)	0.229	-0.408	8.45×10^−6^*
		16	36,220,138	BICF2P1229299	0.091 (0.281/0.041)	0.213	-0.453	1.91×10^−5^*
		6	10,527,823	BICF2S23125774	0.240 (0.500/0.172)	0.212	-0.330	2.05×10^−5^*
		17	18,275,241	chr17_18275241	0.110 (0.313/0.057)	0.187	-0.377	6.96×10^−5^*
		6	75,622,113	BICF2P481353	0.071 (0.219/0.033)	0.184	-0.505	8.37×10^−5^*
		22	48,747,165	BICF2G630335709	0.494 (0.188/0.574)	0.181	0.239	9.66×10^−5^*
		9	8,460,580	BICF2S23511312	0.130 (0.313/0.082)	0.178	-0.395	1.09×10^−4^*
		24	47,255,337	TIGRP2P322787_rs9139922	0.136 (0.344/0.082)	0.176	-0.344	1.19×10^−4^*
English setters	11 deaf, 39 controls	39	111,315,267	BICF2G6304357	0.220 (0.421/0.154)	0.192	-0.276	1.20×10^−3^

af = allele frequency, beta = effect size, pve = proportion of variance explained

All bp positions are listed in canFam3.1 assembly

^a^Calculated using output of GEMMA with equation pve = 1/(1+(N*(se(beta)^2)/beta^2) where N = sample size, se(beta) = standard error of beta. This equation is simplified from Shim *et al*. (2015) [38]

**Significant association using genome-wide threshold determined from unlinked SNPs

*Suggestive association using threshold determined from unlinked SNPs at the chromosome level

Using the quantitative GWAS design, we also identified suggestive associations, that is, the *P*-values reached chromosome-wide significance calculated on unlinked SNPs. All associations from the quantitative GWAS increased in significance when the unilateral dogs were removed (resulting in a case/control bilaterally deaf vs. control dog analysis), but only one had a change in the actual SNP that was most significant, in the Australian cattle dog analysis, from CFA6 to CFA3. QQ plots and lambda values show that we are underpowered in the sibling-pair and quantitative analyses with all lambda values <0.85 (S1 Table), while the bilaterally deaf vs. controls analyses had lambda values between 0.84 and 1.03 (Fig 2B, S1 Fig). Since the bilaterally deaf vs. controls GWAS had the most statistical power, we focus on the results of these analyses.

Our suggestive associations included 35 SNPs (at 8 different loci) in Australian cattle dogs, 12 SNPs (5 different loci) in North American Dalmatians, 1 SNP in UK Dalmatians, and none in English setters (S2 Table). The most significant SNPs at these loci are shown in Table 1. The most significant SNP in the North American Dalmatians was on CFA30 (*P* = 7.3×10^−6^) and in the UK Dalmatians on CFA38 (*P* = 8.2×10^−5^) (S1 Fig, Table 1). The proportion of phenotypic variance explained for the most significant SNP at these suggestive associations ranged from 0.12 to 0.23, and reached 0.28 for the significant SNP on CFA3 in Australian cattle dogs (Table 1).

Genes in the regions of LD surrounding the significant and suggestive associations were investigated (Fig 2C, S2 and S3 Figs) but only a handful of these were good candidate genes for deafness (S3 Table). Of these genes, only one has been identified as causative for deafness in humans (*DFNA7* and *DFNA49* [21]): *ATPase Na*^*+*^*/K*^*+*^ *Transporting Subunit Alpha 4 (ATP1A4)* on CFA38, which had a suggestive association in UK Dalmatians. Two other genes, *Transformation/Transcription Domain Associated Protein (TRRAP)* and *Potassium Inwardly Rectifying Channel Subfamily J Member 10 (KCNJ10)*, have been associated with deafness in humans [36,37]. These were suggestive associations in Australian cattle dogs and UK Dalmatians, respectively. Although we saw several suggestive associations in the analyses of different breeds, we did not identify any loci in common, and we did not identify any suggestive association on CFA20 in the vicinity of the *MITF* locus.

Since Dalmatians, Australian cattle dogs and English setters all have the *piebald* locus, we did a meta-analysis of all breeds combined, in an attempt to increase statistical power. Our results were not significant for the sibling-pair design (*P* = 2.0×10^−3^). For the quantitative and bilaterally deaf vs. controls GWAS, the significant SNPs were due to the population structure of the UK and North American Dalmatian samples, as described above.

## Discussion

Numerous labs have attempted to identify the genetic cause of pigment-associated deafness, but without success. Cargill (2004) [39] genotyped a kindred of 117 Dalmatians using the 172 microsatellite markers of the Minimal Screening Set 1. He identified maximum LOD scores for deafness with markers Cos15 on CFA17 (LOD = 1.69) and FH2585 on CFA28 (LOD = 1.34). There is one human deafness locus in the Cos15 region of CFA17, namely DFNB9 [40], a deafness locus caused by a recessive mutation in the gene *Otoferlin* (*OTOF)* [41]. *OTOF* was a candidate gene mapped in the dog by the FISH technique, with a location of CFA17q13 [42], but Cos15 is localized in the CFA17q11 region [39], so they are not the same. No similar human deafness locus was identified as being in proximity with FH2585. Also, the LOD scores for the study were not statistically significant compared to the threshold level of 3.0 accepted for human and animal linkage studies.

Rak *et al*. (2003) [42] pursued 20 candidate genes identified as causative for deafness in humans or mice, determining canine chromosomal locations and synteny with human locations by FISH and RH mapping, but did not pursue identification of causative deafness relationships [43]. Sommerlad *et al*. (2010) [7] performed a whole genome screen using a set of 325 microsatellite markers in 50 Australian stumpy-tail cattle dogs and mapped an association with deafness to a marker on CFA10 (maximum linkage peak of -log_10_ p-value = 3.64). Further fine mapping was performed on 93 dogs. The closest recognized gene associated with deafness was *SOX10*, which is mutated in human Waardenburg Syndrome type IVc and plays a role in regulating *MITF*, but sequencing in six hearing, two unilaterally deaf, and two bilaterally deaf dogs did not reveal any disease-associated mutations in *SOX10*.

Kluth and Distl (2013) [44] performed a GWAS on 235 German Dalmatian dogs (157 hearing, 78 deaf) using the Illumina CanineHD microarray to identify quantitative trait loci (QTL) associated with deafness. For the combined cohort they identified deafness-associated single nucleotide polymorphisms (SNPs) on CFA2, 6, 17, 27, 29, and 31. They then divided the cohort into dogs with brown eyes and dogs with blue eyes, based on the recognition that blue-eyed dogs are more likely to be deaf. For brown-eyed dogs they identified associated SNPs on CFA2, 6, 14, 27, and 29, while in blue-eyed dogs the associated SNPs were located on CFA17, 18, 27, and 31. The explanation for the different chromosomal SNPs in brown-eyed vs. blue-eyed dogs was non-allelic heterogeneity. No causative genes were identified based on the findings. Some association P-values reached significance with -log_10_P-values > 5.0, but no associated candidate genes were identified.

In addition to microarray GWAS studies, sequencing studies of several candidate genes in deaf vs. hearing dogs have been performed, resulting in reported elimination of the genes *EDNRB* and *KIT* [45], *MYO15A* [46], *PAX3* [47], *TMC1* and *TMIE* [48], *SILV* [49], and *ESPN*, *MYO3A*, *SLC26A5*, and *USH1C* [50]. However, not all of the listed genes have been eliminated in all three breeds of this study.

It is possible that the GWAS and single gene studies described above were unsuccessful because the authors only looked for genes near identified significant SNPs that had already been identified as associated with deafness in humans or mice. New causative mutations continue to be identified at a regular pace, so that this approach may have blinded investigators to locating the actual causative gene. Recently, published proteomic analyses of protein expression in the rat stria vascularis [21] and in mouse inner hair cells [22] have provided information of gene expression in those two tissues, permitting identification of additional candidate genes for deafness. Gene expression in different cochlear cell types for genes identified as causing deafness in humans is also summarized on the Hereditary Hearing Loss Homepage [40]. In pursuit of target genes, we suggest that the initial focus should be on the strial genes, since pigment-associated deafness is initiated by strial degeneration and then followed by hair cell degeneration. However, it is not at present possible to exclude a gene acting on hair cells as causative for canine deafness.

The objective of the present study was to perform GWAS on DNA samples collected from three piebald dog breeds with a recognized high prevalence of deafness: Dalmatians, Australian cattle dogs, and English setters. We used three GWAS designs: (1) samples collected from sibling pairs (one hearing and one deaf in one or both ears), with the assumption that littermates would have less genomic variability than random subjects, (2) quantitative analysis ignoring sibling relationships, and (3) case-control design of bilaterally deaf dogs vs. control dogs.

We identified one significant (at the genome-wide level) and 14 suggestive (at the chromosome level) associations from our analyses. The significant association was identified in Australian cattle dogs using the GWAS design of bilaterally deaf vs. control dogs. A few of the suggestive associations were identified using the quantitative GWAS design but these all increased in significance using the bilaterally deaf vs. control design. Interestingly, the sibling-pair case/control GWAS design performed worse than the quantitative and bilaterally deaf vs. controls GWAS designs in this study. Inflation factors show that the sibling-pair design was very underpowered. We specifically collected siblings from the same litter for this analysis as a way to control for background relatedness, since each case also has a matching related control, while also enabling a relatively sufficient sample size for each breed. However, it appears that this was unsuccessful, possibly partly due to inadequate sample size, which ranged from 66 in English setters to 164 in North American Dalmatians, but also possibly because the control siblings may carry some genetic variants predisposing to deafness that make it hard to disentangle cases and controls genetically. The quantitative GWAS design had an improvement in power over the sibling-pair design, which could be due to the greater sample size and/or the presence of an extra layer of phenotypic information (the unilaterally deaf dogs were separated from the bilaterally deaf dogs) in the quantitative design. The bilaterally deaf vs. control GWAS design had the most power, even though this involved a decrease in sample size and removal of a layer of phenotypic information from the quantitative GWAS design. This unexpected change in GWAS power is because the bilaterally deaf dogs have accumulated more deafness-predisposing alleles than the unilaterally deaf dogs, as shown by Kluth & Distl (2013) [44]. We compared the genotypes at all 9 associations (significant and suggestive) in bilaterally deaf, unilaterally deaf, and control Australian cattle dogs and found that the bilaterally deaf dogs have a higher average number of risk alleles than the unilaterally deaf dogs, and the difference in mean allele count was significant for 8 of the 9 loci (S4 Fig). Rather than being intermediate in risk allele number, the unilaterally deaf dogs were more similar to that of the control dogs.

None of the associations were located on the same chromosome as the known canine white color locus, *piebald* (*MITF* on CFA20), as also found by other previous studies [7,44,51]. Several interesting genes were identified within a 500 kb region of the identified significant and suggestive associations, including *ATP1A4*, *TRRAP*, and *KCNJ10*, which is involved in maintaining electrochemical gradients across the plasma membrane, a component of histone acetyltransferase complexes, and controlling the flow of potassium ions, respectively. In addition to the genetic risk factors in causing deafness, there are other elements that affect expression, including epigenetic and transcriptomic factors, that may play a role during development. Although investigating these non-genetic factors are beyond the current study, it is important to note that MITF is known to have complex interactions with many different genes and transcription factors.

There were no regions of association in common between the three breeds, and no association was identified when we ran a meta-analysis of all breeds combined. These results support our breed-specific analysis design, and suggest that each breed may have a different genetic driver of pigment-associated deafness, pointing to a complex genetic architecture for this canine disease.

One improvement to our study would be to increase sample sizes to boost statistical power in order for our suggestive associations to reach genome-wide significance. It is unfortunate that the Dalmatians showed a genetic distinction based on geographic source (and that we had a biased sampling scheme), such that we were unable to include all samples in a single analysis, thereby reducing the power available for identifying significant associations in that breed. Indeed, when we performed a GWAS with all Dalmatians, the only significant results were all due to this bias in sampling design from geographically distinct locations. All our GWAS had sample sizes of under 200, and simulations have shown that mapping a complex trait with these numbers can be unproductive (see [52]).

Our study shows evidence that congenital pigment-associated deafness in these three piebald breeds is a complex trait but we did not identify any association signal near the *piebald* locus, *MITF*. We find an increase in GWAS power when unilaterally deaf dogs are excluded, suggesting this is a complicated developmental trait that warrants further study with larger sample sizes to disentangle the genetic underpinnings.

## Supporting information

S1 Fig Manhattan and QQ plots for bilaterally deaf vs. control Dalmatian GWAS. A) North American Dalmatians, B) UK Dalmatians. Red line shows the Bonferroni correction *P*-value threshold calculated on unlinked SNPs. Blue line shows the permutation threshold, based on 10,000 random phenotype permutations. Inflation factor (λ) is shown on the QQ plots.
(TIF)

S2 Fig LD plots of the regions surrounding the bilaterally deaf vs. control Australian cattle dog suggestive associations. A) CFA3:17, B) CFA16:36, C) CFA6:10, D) CFA17:18, E) CFA6:75, F) CFA22:48, G) CFA9:8, H) CFA24:47.
(TIF)

S3 Fig LD plots of the regions surrounding the bilaterally deaf vs. control Dalmatian suggestive associations. A) CFA30:37, B) CFA30:33, C) CFA23:48, D) CFA30:22, and E) CFA37:27 in North American Dalmatians. F) CFA38:21 in UK Dalmatians.
(TIF)

S4 Fig Average number of risk alleles in Australian cattle dogs of different phenotypes at the nine significant and suggestive loci. Blue = bilaterally deaf (n = 16), orange = unilaterally deaf (n = 42), grey = control (n = 61). Asterisks show significance (p<0.05) using an unpaired, one-tailed t-test between the number of risk alleles in the bilaterally deaf and unilaterally deaf dogs.
(TIF)

S1 Table Most significant SNPs for quantitative and sibling-pair GWAS designs in Australian cattle dogs, Dalmatians, and English setters. A) Quantitative GWAS, B) Sibling-pair case/control GWAS.
(XLSX)

S2 Table Most significant SNPs from case-control (bilaterally deaf vs. controls) GWAS. Chromosome, bp location, allele frequency, effect size and *P*-value from GEMMA output A) Australian cattle dogs, B) North American Dalmatians, C) UK Dalmatians, D) English setters. ** indicates significant association (at the genome-wide level), * indicates suggestive association (at the chromosome level).
(XLSX)

S3 Table Deafness genes, genes associated with deafness, and possible candidate genes, located within 500 kb of the significant and suggestive SNPs from the bilaterally deaf vs. controls GWAS. (XLSX)
