# Supplementary figures and images for: A genome-wide association study of deafness in three canine breeds
Source: PLoS One. 2020 May 15;15(5):e0232900. doi: 10.1371/journal.pone.0232900 (PMC7228063; doi:10.1371/journal.pone.0232900)

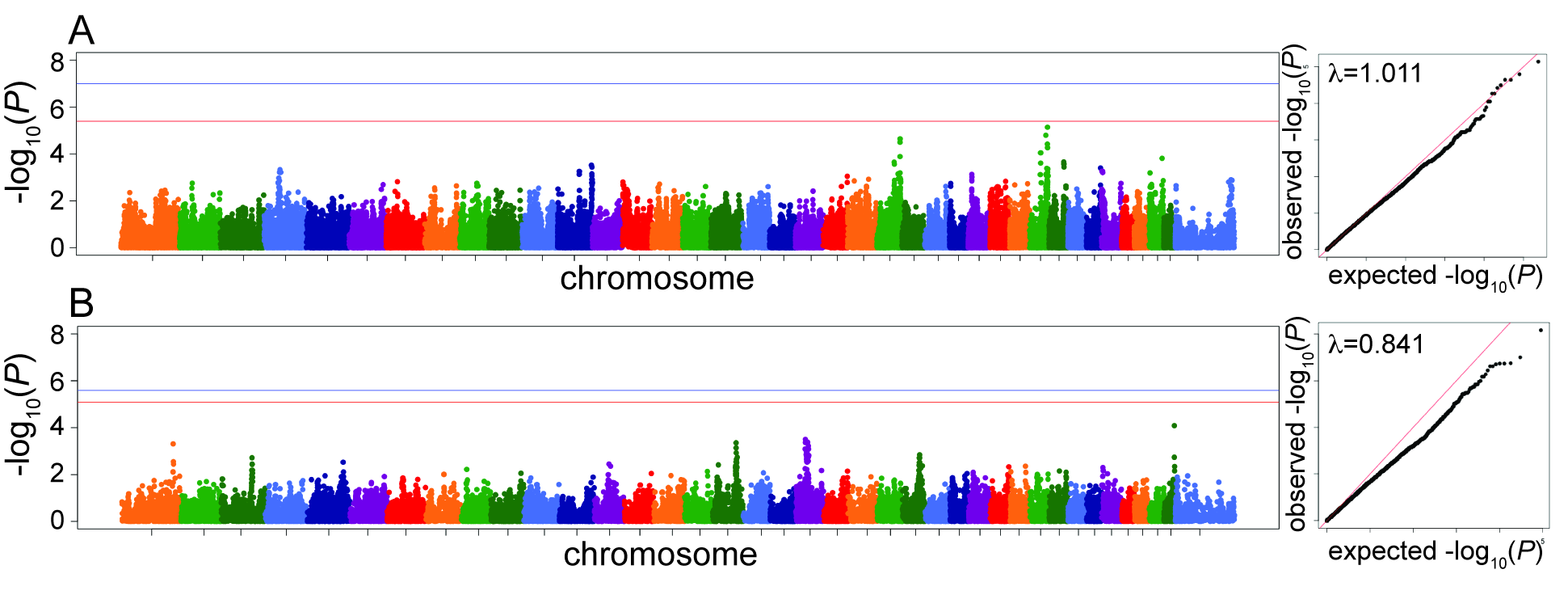

Supplement: S1 Fig — A) North American Dalmatians, B) UK Dalmatians. Red line shows the Bonferroni correction P-value threshold calculated on unlinked SNPs. Blue line shows the permutation threshold, based on 10,000 random phenotype permutations. Inflation factor (λ) is shown on the QQ plots. (TIF) [file pone.0232900.s001.tif]

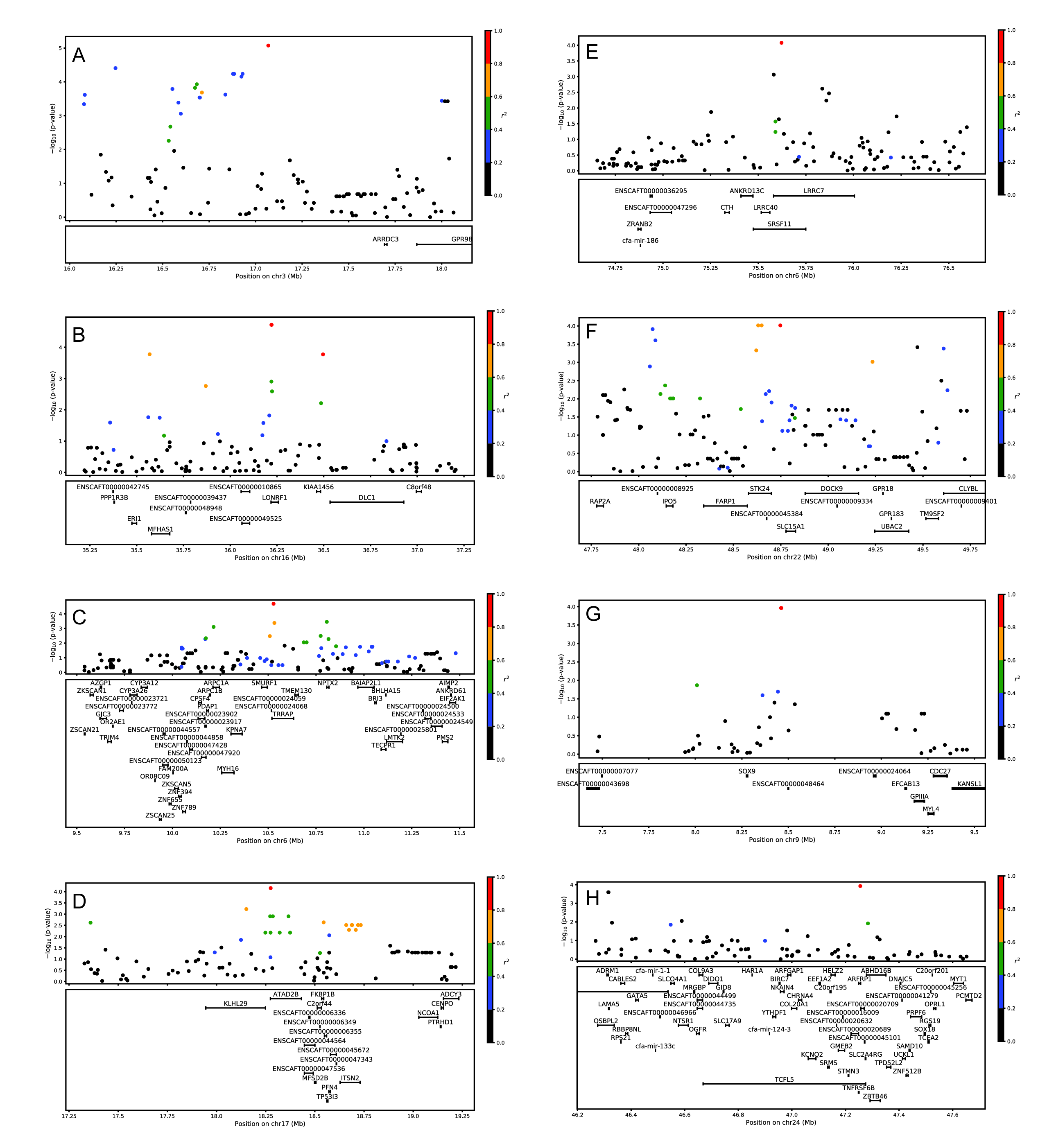

Supplement: S2 Fig — A) CFA3:17, B) CFA16:36, C) CFA6:10, D) CFA17:18, E) CFA6:75, F) CFA22:48, G) CFA9:8, H) CFA24:47. (TIF) [file pone.0232900.s002.tif]

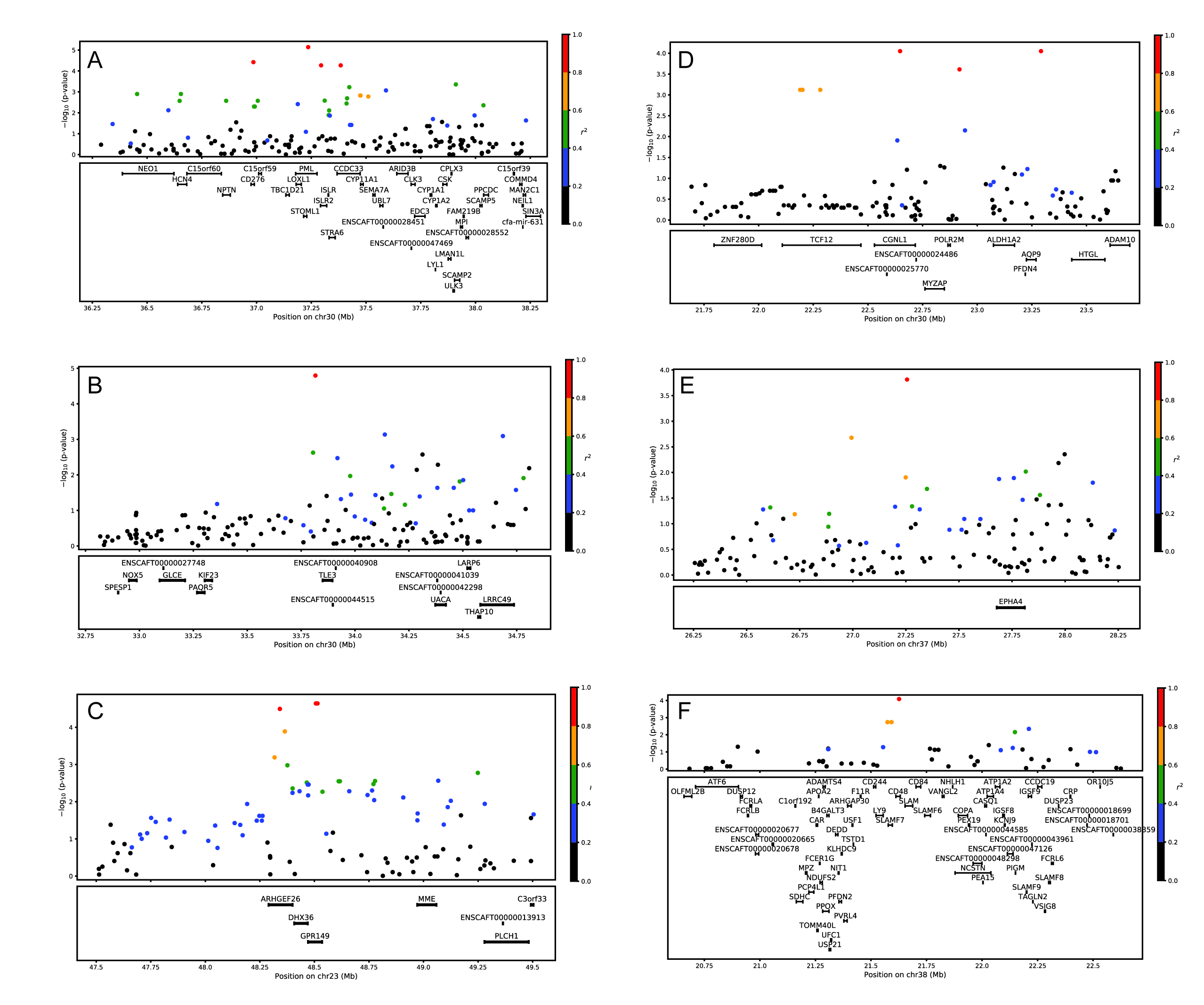

Supplement: S3 Fig — A) CFA30:37, B) CFA30:33, C) CFA23:48, D) CFA30:22, and E) CFA37:27 in North American Dalmatians. F) CFA38:21 in UK Dalmatians. (TIF) [file pone.0232900.s003.tif]

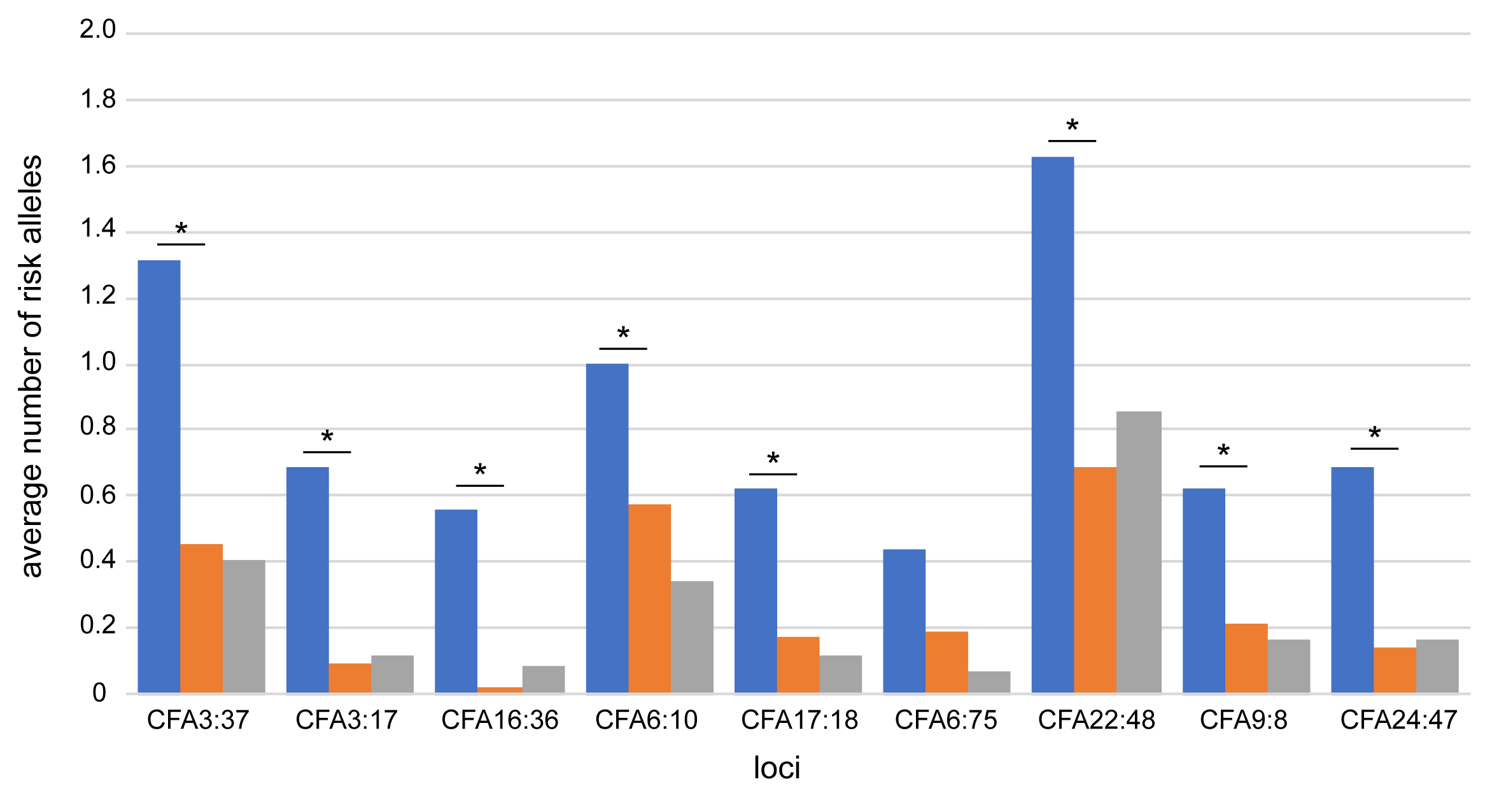

Supplement: S4 Fig — Blue = bilaterally deaf (n = 16), orange = unilaterally deaf (n = 42), grey = control (n = 61). Asterisks show significance (p<0.05) using an unpaired, one-tailed t-test between the number of risk alleles in the bilaterally deaf and unilaterally deaf dogs. (TIF) [file pone.0232900.s004.tif]
